# Entropy-Driven Molecular Beacon Assisted Special RCA Assay with Enhanced Sensitivity for Room Temperature DNA Biosensing

**DOI:** 10.3390/bios14120618

**Published:** 2024-12-15

**Authors:** Shurui Tao, Yi Long, Guozhen Liu

**Affiliations:** 1 CUHKSZ-Boyalife Regenerative Medicine Engineering Joint Laboratory, School of Medicine, The Chinese University of Hong Kong, Shenzhen 518172, China; shuruitao@link.cuhk.edu.cn (S.T.); yi.long01@hotmail.com (Y.L.); 2 Integrated Devices and Intelligent Diagnosis (ID2) Laboratory, Ciechanover Institute of Precision and Regenerative Medicine, School of Medicine, The Chinese University of Hong Kong, Shenzhen 518172, China

**Keywords:** rolling circle amplification, minimum secondary structured RCA, molecular beacon, HPV detection, sensitivity

## Abstract

The Phi29 DNA polymerase is renowned for its processivity in synthesizing single-stranded DNA amplicons by rolling around a circularized DNA template. However, DNA synthesis rolling circle amplification (RCA) is significantly hindered by the secondary structure in the circular template. To overcome this limitation, an engineered circular template without secondary structure could be utilized to improve the sensitivity of RCA-based assays without increasing its complexity. We herein proposed an entropy-driven special RCA technology for the detection of HPV16 E7 gene at room temperature. The strategy is composed of a molecular beacon containing a loop region for nucleic acid target recognition and a stem region to initiate RCA. With the target analyte, the stem region of the molecular beacon will be exposed and then hybridized with a special circular template to initiate the DNA amplification. We tested different designs of the molecular beacon sequence and optimized the assay’s working conditions. The assay achieved a sensitivity of 1 pM in 40 min at room temperature. The sensitivity of this assay, at 1 pm, is about a hundred-fold greater than that of conventional linear RCA performed in solution. Our proposed sensor can be easily reprogrammed for detecting various nucleic acid markers by altering the molecular beacon’s loop. Its simplicity, rapid assay time, and low cost make it superior to RCA sensors that utilize similar strategies.

## 1. Introduction

Nucleic acids are a type of biopolymer which is a fundamental component for storing, encoding, transmitting, and expressing genetic information in living organisms. Due to the unique nucleic acid sequences in each species, nucleic acids have become a crucial factor in classifying biological populations [1,2]. Nucleic acid amplification techniques (NAATs), including polymerase chain reaction (PCR), loop-mediated isothermal amplification (LAMP), rolling circle amplification (RCA), recombinase polymerase amplification (RPA), nucleic acid sequence-based amplification (NASBA), and exponential amplification reaction (EXPAR), are widely used in both scientific research and clinical settings. Each method provides distinct benefits regarding sensitivity, specificity, ease of use, stability, and cost-effectiveness. NAATs are especially important in molecular diagnostics, particularly for point-of-care applications where there is a considerable unmet demand [3].

RCA is an effective and straightforward technique for isothermal enzymatic nucleic acid amplification. Utilizing a circular template and a DNA or RNA primer, RCA can achieve detection of the target in low concentration and exceptional specificity under isothermal conditions [4]. Linear RCA (L-RCA) is the basic form of all RCA-derivative technologies, which includes only a single primer and a corresponding circular template for amplification [5]. Normally, L-RCA cannot achieve high sensitivity, as the amplification efficiency of the L-RCA is about 10^3^ copies, which is not advantageous in detecting low-abundance markers [6,7]. Thus, recent research mainly focuses on feedback processes to produce exponential RCA (E-RCA); many methods were developed for exponential RCA for higher sensitivity. Hyperbranched-RCA (H-RCA) is realized by designing one or more primers that are complementary to the L-RCA amplification product. By extending these primers in place of each other, a very high amplification efficiency can be obtained, increasing the sensitivity by four orders of magnitude; Nilson and colleagues hybridized an oligonucleotide to the RCA product to form a double strand, which was then digested with a restriction endonuclease, followed by a template-mediated enzymatic linkage reaction to generate a number of new circular DNAs that could be used as templates for the next amplification step with a second set of primers [8]. RCA techniques were also combined with other enzymes to improve the sensitivity; Xu et al. introduced nickase to generate nicked fragments from L-RCA amplicons continuously, and their system was able to detect a minimum concentration of 5 pM of miRNA [9]. Nicking endonuclease-mediated RAC could be further combined in one pot with hairpin assembly to achieve real-time fluorescence detection of nucleic acids [10]. These coupled methodologies enhance the assay’s sensitivity but increase its complexity; considerable user intervention is required to control reaction timing. Researchers have also developed an RCA-based method using only one DNA polymerase for simplicity. For example, Kim et al. used a dumbbell probe and gold nanoparticles to achieve a one-step detection of miRNA [11]; Tian et al. performed RCA on carbon nanotubes to enhance the electron transfer, achieving a sensitivity of 1.2 fM [12]. Huang et al. proposed a toehold-mediated feedback rolling circle amplification (TMFRCA) strategy; the RCA amplicons react with toehold probes to initiate new rounds of RCA [13]. However, they exhibit low sensitivity, require labeling probes, or involve the use of multiple DNA probes and other chemicals, which complicates the reaction.

The secondary structure of the RCA amplicons is most likely the reason for the lower synthesis rate. As a result, RCA requires a longer reaction time (up to several hours). We previously demonstrated that carefully engineering the DNA circular template could greatly enhance the sensitivity of RCA-based assays. An engineered circular template with a repeating sequence that could generate RCA amplicons with minimum secondary structure, namely minimum structured rolling circle amplification (MSSRCA), has a hundred-fold superior sensitivity than traditional RCA in its linear amplification mode; however, the restriction in the design of the circular template hindered its usage in specific nucleic acid target detection [14]. We further incorporated a specific nicking enzyme recognition site into the recognition probe for the nucleic acid target; the reaction cascade produces primers for the MSSRCA by cleavage of the nicking enzyme on the DNA duplex [15].

With its inherent stability, specificity, and simplicity, molecular beacon-assisted isothermal DNA amplification technology has recently become a possible method for quick and affordable oligonucleotide detection [16,17]. When recognized and hybridized to a complementary nucleic acid molecule, the stem-loop structure can be disrupted for an exposed primer to initiate RCA. We demonstrated here that our proposed method has the advantage of being stable, efficient, label-free, and cost-efficient with only one enzyme mediated in the reaction. Since the oncogenic mRNA expression levels of high-risk HPV have been confirmed to strongly correlate with cancer development, oncogenic mRNA has the potential to act as a significant biomarker for detecting high-risk HPV infections that may progress, thereby serving as an important tool for patient screening and management [18,19]. We herein report a method that linked MSSRCA with HPV16 E7 gene detection with the help of a molecular beacon. The entropy-driven hybridization of the molecular beacon with the target and the conformation change of the molecular beacon allows the MSSRCA to proceed. Our method achieved rapid, simple, and room temperature detection of E7 mRNA and has a potential application in point-of-care (POC) settings.

## 2. Reagents and Materials

Phi29 DNA polymerase and ssDNA/RNA CircLigase with their respective buffers were acquired from HaiGene Biotech (Harbin, China). Ethanol ≥ 99.5% was purchased from Aladin Scientific and diluted using Milli-Q water (Shanghai, China). The 6× DNA loading buffer (#D2041) for PAGE was purchased from US Everbright Inc. (Jiangsu, China). SYBR Gold Nucleic Acid Gel Stain (10,000× in DMSO) was sourced from Thermo Fisher (Shanghai, China), while SYBR Green I (#S171397) was obtained from Aladdin (Shanghai, China); fluorescence dye was further diluted using Milli-Q water and stored at −20 °C for short-term usage. DNA ladders (20 bp and 15,000 bp) were procured from Takara company (Beijing, China). The deoxynucleotide triphosphates (dNTPs) mixture (#PC2300) was purchased from Solarbio (Beijing, China). The Sangon Oligos DNA Purification Kit (#B511143) was procured from Sangon (Shanghai, China) for the purification of gel products. Oligonucleotides used in this study were synthesized and purified using high-performance liquid chromatography (HPLC) at Shanghai Sangon (Table S1). Throughout the experiments, all reagents were employed as received without the need for additional purification steps. Deionized water (18.2 MΩ) was sourced from a Milli-Q water purification system for all experiments. Room temperature (RT) refers to 22 °C. The buffers utilized in this study include the following: 1× Phi29 buffer: 50 mM Tris-HCl, 10 mM MgCl_2_, 10 mM (NH4)_2_SO_4_, 4 mM DTT, pH 7.5; Diffusion buffer: 0.5 M ammonium acetate, 10 mM magnesium acetate, 1 mM EDTA, pH 8.0, 0.1% SDS; 1× circ ligase buffer: 3 mM Tris-acetate, 6 mM potassium acetate, 1 mM DTT, pH 7.5; 1× Phi29(2) buffer: 33 mM Tris-acetate, 10 mM Mg-acetate, 66 mM K-acetate, 0.1% of (*w*/*v*) Tween 20, 1 mM DTT pH 7.9; and 1× NEB2.0 buffer: 50 mM NaCl, 10 mM Tris-HCl, 10 mM MgCl_2_, 1 mM DTT, pH 7.9.

### 2.1. Apparatus

The concentrations of DNA and protein were determined using Thermo Scientific™ NanoDrop™ One Microvolume UV-Vis Spectrophotometer according to the instructions. Fluorescence measurements were taken with a PerkinElmer EnVision (Waltham, MA, USA) multimode or ID5 microplate reader (San Jose, CA, USA). Agarose gel electrophoresis and PAGE were conducted using a Bio-Rad Power pack with a constant voltage electrophoresis system (Waltham, MA, USA) and imaged using either a Jiapeng JP-BL100 blue-light transilluminator (Shanghai, China) or a TIANGEN TGel Image System (Beijing, China).

### 2.2. Circularization of ssDNA to Make a Circular Template

The circularization reaction contained 1× Ligation buffer, 6.25 mM MnCl_2_, 50 µM ATP, and 5 µM of the circular template. The final volume was brought to 20 µL. The reaction was performed at 60 °C for 60 min and then brought to 80 °C to inactivate the CircLigase. The product was characterized by 15% denaturing urea polyacrylamide gel electrophoresis.

### 2.3. Page Gel Electrophoresis of the MSSRCA Circular Templates

To prepare the DNA sample, 10 µL of DNA was combined with an equal volume of formamide and heated to 95 °C for 10 min. Subsequently, 5 µL of the 6× PAGE loading buffer was introduced into the mixture. The prepared sample was then applied onto a 15% denaturing urea-polyacrylamide gel and subjected to electrophoresis at 100 V for 1 h at 55 °C. Post-electrophoresis, the gel was stained using a 1× SYBR Green I solution diluted in Milli-Q water.

### 2.4. Extraction of Circular Templates from Urea-PAGE Gel

The protocol is based on our previous publication [14]. Briefly, the ligation process involved creating the control and ligase samples by adding 5 µL of the 10× Ligase buffer and 10 µL of the 100 µM ligase as per the manufacturer’s instructions. The ligation mixture was incubated at 60 °C for 2 h and 95 °C for 10 min. Subsequently, purification through PAGE involved preparing a 7M urea gel, incubating samples at 95 °C with formamide, pre-running the gel, loading samples with the loading buffer, and running the gel. Gel fragments containing DNA segments were excised, crushed, and subjected to diffusion buffer incubation and centrifugation before storage at −20 °C. Ethanol precipitation was performed by adding ethanol to the suspension, incubating, centrifuging, washing, and air drying. Further purification utilized a Sangon UNIQ-10 DNA kit, involving binding, washing, and elution steps. Quality control was conducted by analyzing the purified circular templates using a NanoDrop UV-VIS for spectral scanning, with a peak around 260 nM indicating successful purification.

### 2.5. Standard Protocol for Molecular Beacon-Assisted MSSRCA

Solution A included the following: 10.75 µL of ddH_2_O, 2 µL of 50 nM of molecular beacon, 2 µL of 10× NEB2.0 buffer (50 mM NaCl, 10 mM Tris-HCl, 10 mM MgCl2, and 1 mM DTT), and 2 µL of different amount of target. Solution B included the following: 0.25 µL of Phi29 polymerase (10 U/µL), 1 µL of 10 mM dNTPs, and 2 µL of 100 nM circular template. Solution A was first heated to 95 °C for 5 min and placed at RT for 10 min before the addition of Solution B. The mixture was then incubated at RT for 40 min unless otherwise mentioned.

### 2.6. Fluorescence Measurement

DNA products were stained with a final concentration of 2× SYBR Gold. Fluorescence measurements were conducted using a PerkinElmer EnVision multimode plate reader, with excitation and emission wavelengths set to 495 nm and 537 nm, respectively.

## 3. Result and Discussion

### 3.1. Principle of the Molecular Beacon-Assisted MSSRCA

**Scheme 1:**
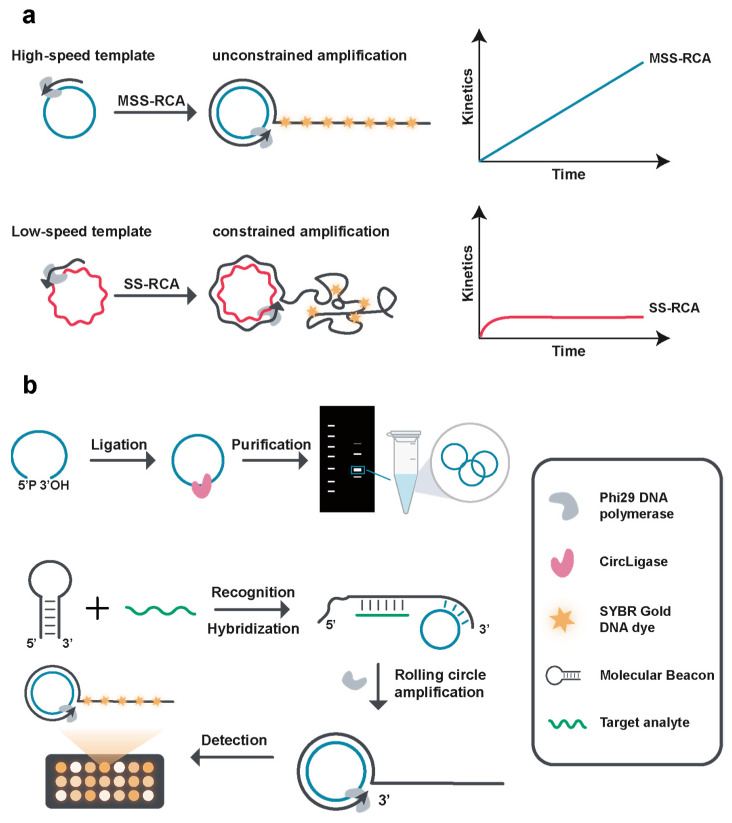
Principle of the molecular beacon-assisted entropy-driven MSSRCA for room temperature nucleic acid detection. The (**a**) comparison between the MSSRCA and the SSRCA is shown, and the MSSRCA benefits from the minimum secondary structured circular template used, resulting in high-speed, unconstrained amplification using Phi29 DNA polymerase. The (**b**) scheme using our assay for nucleic acid detection is shown, and the special circular template is first ligated using CircLigase and purified from PAGE. In the one-tube reaction, the molecular beacon undergoes a conformational change upon target gene binding, allowing the circular template to hybridize and initiate the MSSRCA process. Phi29 DNA polymerase then extends the molecular beacon, forming a linear DNA strand. SYBR Gold dye intercalates with the DNA, providing fluorescence to monitor MSSRCA amplicon generation.

The principle for our molecular beacon-assisted MSSRCA assay is shown in Scheme 1. Our one-step nucleic acid detection platform comprises a molecular beacon with a hairpin structure, a specially engineered circular template, and the Phi29 DNA polymerase. A conformational change occurs upon binding the target gene to the molecular beacon’s loop region. This activation allows the circular template to match and hybridize with the end of the molecular beacon, thereby initiating the MSSRCA process. The polymerase will continuously add dNTPs to the 3′ end of the molecular beacon to form a linear DNA strand since the circular template is carefully designed. SYBR Gold is a fluorescence dye that intercalates to the DNA strand and elicits strong fluorescence [20] and is therefore applied in our system to monitor the generation of MSSRCA amplicons. Consequently, the fluorescence intensity correlates directly with the MSSRCA product, establishing a relationship between the target concentration and the corresponding fluorescence intensity.

The design of the molecular beacon sequence is crucial for effective target detection. It is essential that the presence of the target mRNA leads to the unwinding of the beacon probe, resulting in the separation of its stem. Conversely, when the target nucleic acid is absent, the molecular beacon retains its closed stem-loop structure, thereby inhibiting the initiation of MSSRCA. The molecular beacon probe in this assay comprises a 20 bp stem region and a 32 nt target hybridization site. Additionally, the primer for MSSRCA is 13 nt long and located at the 3′ end within the enclosed stem of the molecular beacon (Figure S1A,B).

### 3.2. Feasibility of the Assay

The link between HPV infection and cervical cancer (CC) is well established, with more than 99.7% of CC cases containing HPV DNA. HPV strains 16 and 18 are predominantly accountable, accounting for nearly 70% of all CC cases [21,22]. These high-risk HPV types promote malignant transformation by establishing persistent infections that result in the overexpression of the viral oncogenes E6 and E7. The cellular activity of E6 and E7 oncoproteins with crucial cell cycle regulatory disruptions highlights their essential role in tumorigenesis [23,24,25].

**Figure 1:**
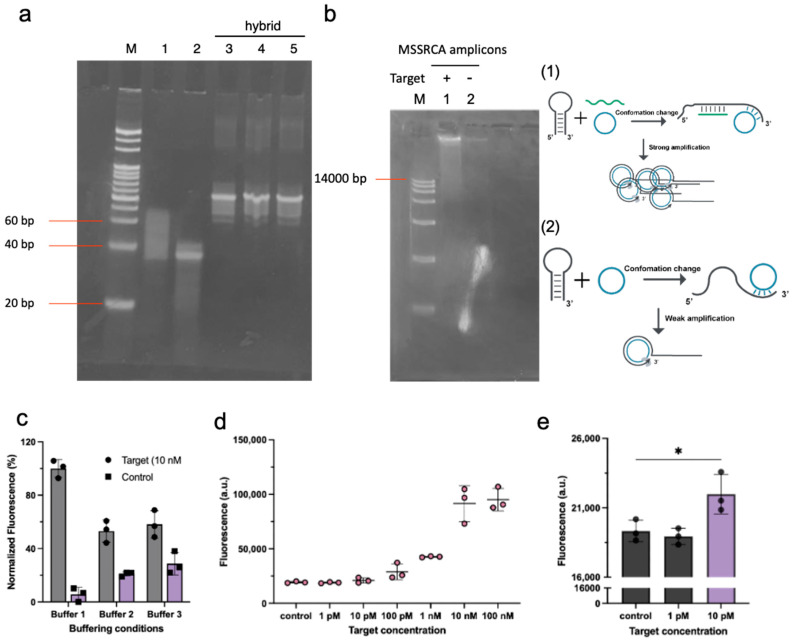
**Feasibility of the nucleic acid sensing.** (**a**) Hybridization of the molecular beacon and target (Lane 1: synthetic E7 DNA; Lane 2: molecular beacon 1; Lane 3: hybridization in 1× Phi29 buffer; Lane 4: hybridization in 1× NEB 2.0 buffer; and Lane 5: hybridization in 1× Phi29 (2) buffer) is shown. For each experiment, a 5 µM of concentration was used for all probes. (**b**) Agarose gel electrophoresis of the molecular beacon-assisted MSSRCA in the presence or absence of a 10 nM HPV16 E7 DNA is shown. (**c**) Optimization of the buffering conditions for the assay is shown (Buffer 1: 1× NEB 2.0 buffer; Buffer 2: 1× Phi29 buffer; and Buffer 3: 1× Phi29 (2) buffer). The fluorescence intensity is normalized within groups. (**d**) Calibration curve of the molecular beacon-assisted MSSRCA using molecular beacon 1 is shown. (**e**) Sensitivity of the molecular beacon-assisted MSSRCA using molecular beacon 1 is shown. *n* = 3, and error bars represent means  ±  s.d. Two-tailed *t*-test was performed using GraphPad prism software Version 9.5.0 with 0.1234 (ns), 0.0332 (*).

We used a synthetic HPV16 E7 gene (GenBank: MK343362.1) to test the feasibility of the assay. We first performed non-denaturing gel electrophoresis to confirm the conformational change of the molecular beacon. Lane 3, lane 4, and lane 5 are the hybrids of the molecular beacon and the synthetic gene in 1× Phi29 buffer, 1× NEB 2.0 buffer, and 1× Phi29 (2) buffer, respectively (Figure 1a). Regarding the molecular beacon, an E7 DNA duplex was perfectly found upon the above buffering conditions with a slower migration speed. We next performed agarose gel electrophoresis of the MSSRCA assay with and without the addition of 10 nM synthetic E7 DNA. The presence of 10 nM of the target led to a substantial increase in the formation of MSSRCA amplicons; as shown in Figure 1b, the gel band in lane 1 is considerably more intense than that in lane 2. Theoretically, the MSSRCA product should not have been observed when E7 DNA is absent in the reaction. It is, however, known that the molecular beacon itself will undergo phase transitions in the solutions [26,27], which means that a small amount of MSSRCA products is likely to have originated from the direct hybridization between the molecular beacon and the circular template. We tested the reaction with three different buffers that are suitable for the Phi29 polymerase. As shown in Figure 1c, the NEB 2.0 buffer has a higher signal-to-noise ratio; we then used the NEB 2.0 buffer for the reaction. We carried out the calibration curve of the assay; we found that the sensitivity of the system in one hour of the reaction time is 10 pM (Figure 1d,e). The sensitivity is superior to other RCA-based assays in linear amplification modes [6]. To further improve the sensitivity of the system, we carried out optimization experiments in the design of the beacon, ion concentration, time intervals, and amount of reagents.

### 3.3. Optimization of the Experimental Conditions

**Figure 2:**
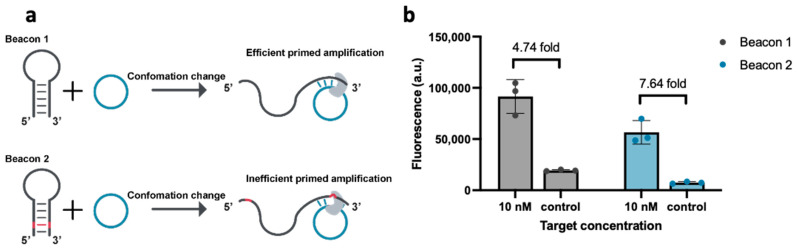
**Optimization of the design of the molecular beacon.** (**a**) Schematic illustration of the mismatch primed MSSRCA. (**b**) Comparison of the fluorescence signal using two different molecular beacons.

We first introduced one mismatch to the stem of the molecular beacon (Figure 2a). Functions of the one mismatch include the following: (1) increase the stability of the closed stem of the molecular beacon and (2) slow down the hybridization between the circular template and the stem. Since the Phi29 polymerase has the 3′ to 5′ exonuclease activity, it can correct the mismatch and finally convert the molecular beacon to an MSSRCA primer [28,29]. The mismatch decreased the amplification efficiency but increased the detection efficiency by lowering the background signal (Figure 2b). We used the Mfold web server to predict the molecular kinetics of the two designs. As shown in Table 1, the hybridization energy for the stem of the beacon and beacon/target hybrid does not change much with the mismatch. However, the hybridization tendency between the molecular beacon and the circular template is decreased.

**Figure 3:**
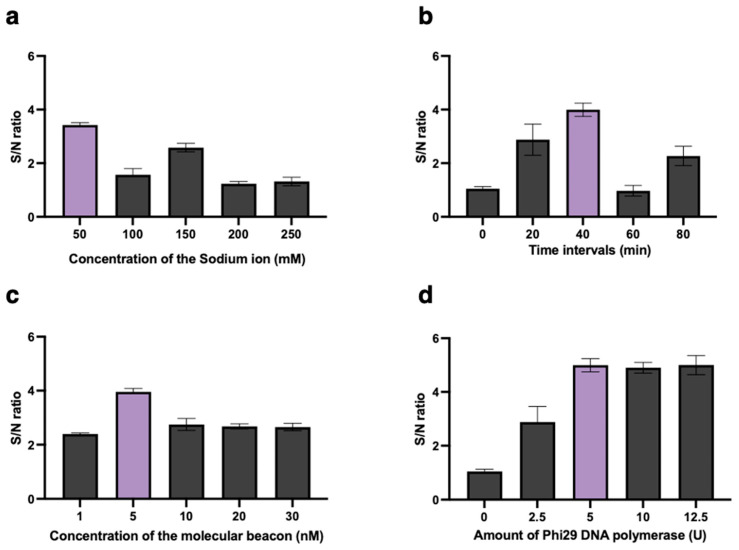
**Optimization of the molecular beacon assisted MSSRCA.** Optimization of (**a**) sodium ion concentration, (**b**) time intervals, (**c**) concentration of the molecular beacon, and (**d**) amount of Phi29 DNA polymerase using 1 nM of the target nucleic acid.

This study optimized the experimental parameters to achieve optimal sensing performance further. Sodium ion concentration will greatly affect the stability of the molecular beacon. As shown in Figure 3a, as the sodium ion concentration gradually increases, the background noise is significantly lowered, the enzyme activity is also hampered, thus affecting the overall performance of the assay. The assay time was fixed at 40 min because the circular template hybridizes with the molecular beacon and initiates the amplification at a lower rate than in the presence of the target. Consequently, after 40 min, the signal-to-noise ratio decreased significantly (Figure 3b). The amount of the molecular beacon is a key factor in its relation to the background signal. In this assay, the circular template can also hybridize with the molecular beacon in dynamics, so an excess amount of molecular beacon will cause a high background signal. As shown in Figure 3c, when 1 nM of the target was challenged with different amounts of molecular beacon, 5 nM of molecular beacon obviously increased the S/N ratio. Therefore, a concentration of 5 nM of the molecular beacon was determined to be optimal for inclusion in the assay. As depicted in Figure 3d, the impact of varying the quantity of Phi29 DNA polymerase was examined. It was evident that the S/N ratio notably rose as the molar of Phi29 DNA polymerase increased, peaking at 5 U. Consequently, 5 U of the polymerase was identified as an optimal amount of Phi29 polymerase for the study.

### 3.4. Analytical Performance of the Assay

**Figure 4:**
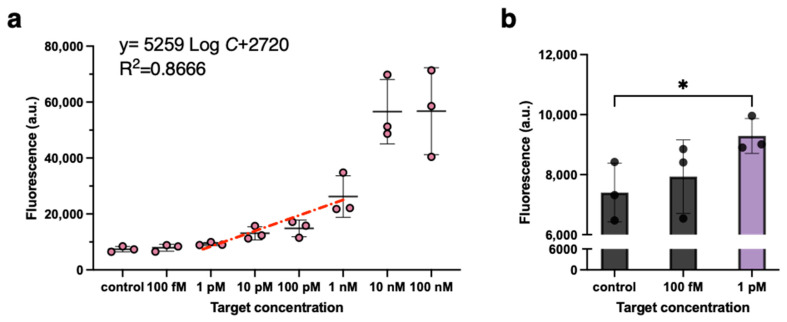
**Detection of HPV16 E7 DNA using molecular beacon-assisted MSSRCA.** (**a**) shows the target-induced fluorescence response of the system. The target concentration of 100 fM to 100 nM was tested. n = 3, and the bars represent means  ±  s.d. (**b**) shows fluorescence of the reaction using the nucleic acid target concentration of 100 fM and 1 pM. A two-tailed t-test was performed using GraphPad Prism software Version 9.5.0 with 0.1234 (ns), 0.0332 (*).

The sensitivity of the molecular beacon-assisted MSSRCA for detecting HPV16 E7 was studied under optimal conditions. We measured the endpoint fluorescence with different target concentrations. The fluorescent signal generated from each reaction diminished in proportion to the concentration of the DNA trigger, indicating that molecular beacon-assisted MSSRCA is effective for quantifying variations in DNA levels (Figure 4). The fluorescence exhibits a log-linear regression relationship with the target’s concentrations ranging from 1 pM to 1 nM, demonstrating the ability of our system to detect nucleic acids quantitatively. The sensor achieved the lowest detection threshold of 1 pM in 40 min under room temperature based on the two-tailed t-tests [30]. This result underscores the significant advantage of MSSRCA in terms of synthetic kinetics and its feasibility at room temperature, indicating that the rate of the DNA polymerization reaction is faster and less affected by temperature fluctuations.

## 4. Discussion

We are developing a new isothermal amplification and detection strategy for sensitive and rapid nucleic acid quantification. This was accomplished by engineering the secondary structure of the RCA amplicons to enhance DNA amplification and connect the target gene with the MSSRCA process using a molecular beacon. This work, for the first time, combined the molecular beacon with the novel MSRCA technique. This assay exhibited a more than fifty-fold increased sensitivity than traditional linear RCA [31] in one tube under room temperature that has a limit of detection of 1 pM. We also provided a general strategy in the design of the molecular beacon, which incorporates both polymerization and exonuclease functions of the Phi29 DNA polymerase.

By employing a rational design for the molecular beacon, this assay has the capacity to detect a variety of cancer-related nucleic acid biomarkers. Although an adequate detection limit was not achieved for HPV oncogenic mRNA using this system, as a proof-of-concept, this enables the expansion of RCA-based sensors in point-of-care applications. This method could be accommodated for markers with higher abundance where only moderate enhancements in the limit of detection are required, such as miR-21 in cancer cells.

We highlight that the assay offers a target-to-result time of 40 min; we expect improvements in the sensing performance with better sequence design [32], integration of nanomaterials [33] paper device [34], and incorporation of multi-primed RCA [35]. We also expect improvements in the sensitivity with cross-amplification by implementing various feedback processes.

## Figures and Tables

**Table 1:** Comparison between different designs of molecular beacons.

	Tm (°C)	ΔG (kcal/mol)	ΔH (kcal/mol)	ΔS (kcal/mol/K)
Beacon 1 stem	61.8	−18.7	−145	−407.3
Beacon 2 stem	63.7	−19.5	−140.7	−408.1
Beacon 1 + circular template	56.8	−17.2	−154.9	−443.8
Beacon 2 + circular template	51	−14	−140.3	−407.3
Beacon 1 + target	82.1	−41.3	−263	−714.8
Beacon 2 + target	82.1	−41.3	−263	−714.8

## Data Availability

Data are contained within the article.

## Supplementary Materials

The following supporting information can be downloaded at: https://www.mdpi.com/article/10.3390/bios14120618/s1, Table S1: The sequences used in this study; Figure S1: Design of the molecular beacon. (a) self-folding of the molecular beacon 1. (b) hybridization of the molecular beacon and the E7 gene; Figure S2: Mismatch introduced to the molecular beacon 2.
