# Supplementary material for: Entropy-Driven Molecular Beacon Assisted Special RCA Assay with Enhanced Sensitivity for Room Temperature DNA Biosensing
Source: Biosensors (Basel). 2024 Dec 15;14(12):618. doi: 10.3390/bios14120618 (PMC11674589; doi:10.3390/bios14120618)
Supplement: Supplementary file 1 [file biosensors-14-00618-s001.zip › biosensors-3346462-supplementary.pdf]

Article

Entropy-Driven Molecular Beacon Assisted Special RCA Assay with Enhanced Sensitivity for Room Temperature DNA Biosensing

Shurui Tao 1,2, Yi Long 1,2 and Guozhen Liu 1,2,*

1 CUHKSZ-Boyalife Regenerative Medicine Engineering Joint Laboratory, School of Medicine, The Chinese
University of Hong Kong, Shenzhen 518172, China; shuruitao@link.cuhk.edu.cn (S.T.);
yi.long01@hotmail.com (Y.L.)

2 Integrated Devices and Intelligent Diagnosis (ID2) Laboratory, Ciechanover Institute of Precision and
Regenerative Medicine, School of Medicine, The Chinese University of Hong Kong, Shenzhen 518172, China * Correspondence: liuguozhen@cuhk.edu.cn

Design of the sequence

Table S1. The sequences used in this study.

Name	Sequence (5' to 3')
HPV16 E7 gene	AAATGACAGCTCAGAGGAGGAGGATGAAA TAGATGGTCCAGCTGGACAAG
Circular template	GGTTATTATTGGTTATTATTGGTTATTATTG GTTATTATTGGTTATTATT
Primer 1	ATCCTCCTCCTCTGAGCTGTCATTTACCAAT AATAACCAATAATAACCAATAATA
Molecular Beacon 1	GGTTATTATTGGTTATTACTGGACCATCTATT TCATCCTCCTCCTCTGAGTAATAACCAATAA TAACC
Molecular Beacon 2	GGTTAGTATTGGTTATTACTGGACCATCTA TTTCATCCTCCTCCTCTGAGTAATAACCAAT ACTAACC

Underlined: stem of the molecular beacon. Red: complementary to the circular template. Yellow highlight: location of the mismatch.

The sequence of HPV16 E7 mRNA was found at GenBank (GenBank: MK343362.1; https://www.ncbi.nlm.nih.gov/nuccore/MK343362.1?report=fasta). After that, the sequence is pasted to the Mfold for secondary structure prediction[10]. We selected the region with the least secondary structure to maximize assay efficiency[11], and the molecular beacon was designed accordingly. The self-folding and the hybridization folding were checked using Mfold before we finalized the design.

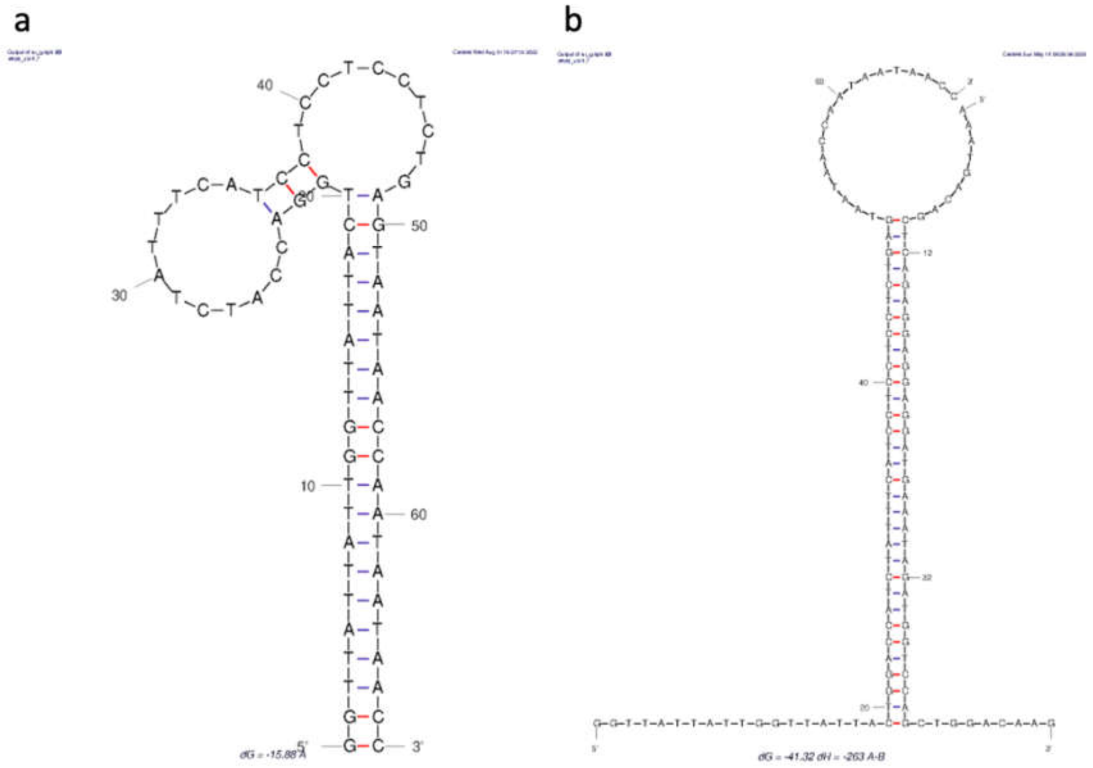

Figure S1. Design of the molecular beacon. (a) self-folding of the molecular beacon 1. (b) hybridization of the molecular beacon and the E7 gene.

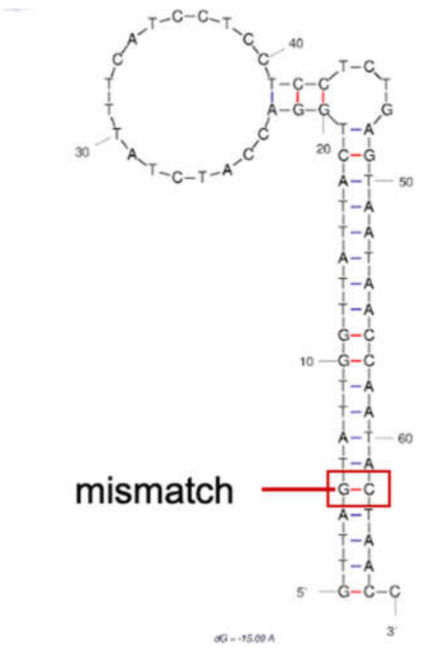

Figure S2. Mismatch introduced to the molecular beacon 2.
